# Successful bailout stenting strategy against lethal coronary dissection involving left main bifurcation

**DOI:** 10.1002/ccr3.972

**Published:** 2017-04-24

**Authors:** Hiroshi Kubota, Tetsuya Nomura, Yusuke Hori, Kenichi Yoshioka, Daisuke Miyawaki, Ryota Urata, Takeshi Sugimoto, Masakazu Kikai, Natsuya Keira, Tetsuya Tatsumi

**Affiliations:** ^1^ Department of Cardiovascular MedicineNantan General HospitalNantan CityKyotoJapan

**Keywords:** Bailout stenting, coronary dissection, iatrogenic, left main bifurcation

## Abstract

Catheter‐induced coronary dissection involving left main bifurcation is a rare complication during cardiac catheterization but can become lethal unless it is treated appropriately. Interventional cardiologists always have to pay attention to the risk of complications related to cardiac catheterization and prepare for determining the best bailout strategy for the situation.

## Introduction

Catheter‐induced coronary artery dissection is an uncommon complication. However, once critical coronary dissection occurs, the outcome can be fatal. The incidence of iatrogenic left main coronary artery (LMCA) dissection is reported to be approximately 0.07% (0.06% of coronary angiography (CAG), 0.10% of percutaneous coronary intervention (PCI)), and almost twice as common with PCI as CAG 1. In the NHLBI classification system for coronary artery dissection types, those of type A and B are clinically benign in general, whereas those of type C through F lead to significant mortality unless they are rapidly and optimally treated 2.

## Case Report

An 82‐year‐old female was admitted to our hospital complaining of repetitive rest angina in the early morning continuing for several months. She had a past medical history of hypertension alone. Her blood pressure was 146/88 mmHg, and pulse was 76/min and regular. Her body mass index was 21.4. Physical examination and laboratory data showed no abnormal finding. Twelve‐lead electrocardiography demonstrated a normal sinus rhythm and no sign of cardiac ischemia. Chest X‐ray showed calcification of the aortic arch and a slight increase in the cardiothoracic rate (51%). Ultrasound echocardiography demonstrated normal left ventricular wall motion and no apparent valvular disorder. We suspected coronary spastic angina pectoris based on her clinical course.

**Figure 1:**
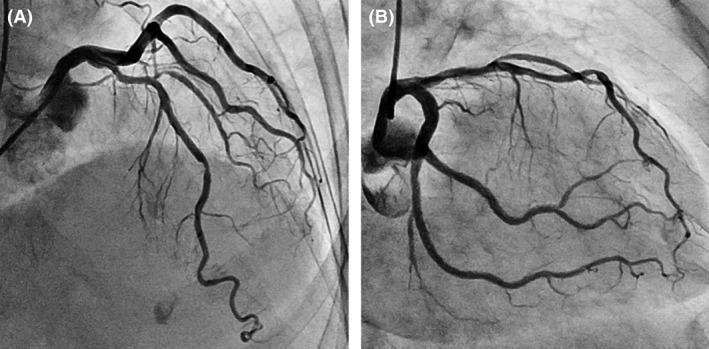
No significant organic stenosis was observed in the left coronary artery. (A) Antero‐posterior and cranial view. (B) Right anterior oblique and caudal view.

**Figure 2:**
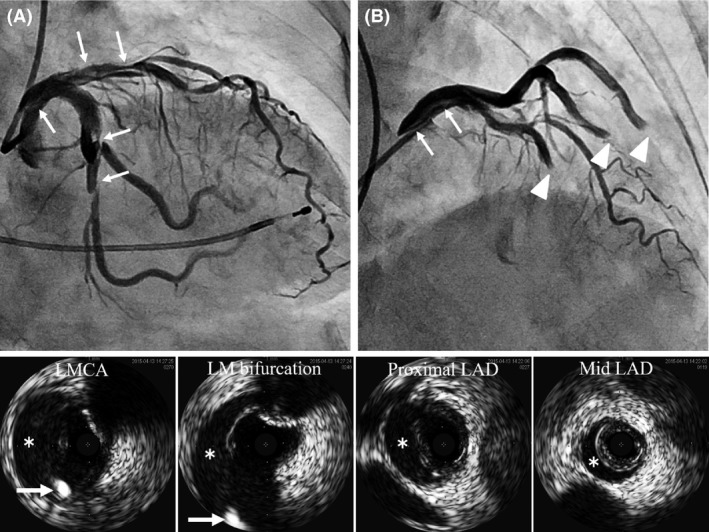
Spiral coronary dissection occurred and rapidly spread out in the three directions of the proximal LMCA, LAD, and LCX coronary arteries (A, B Arrows). Coronary blood flow in both the LAD and LCX coronary arteries was interrupted by a coronary hematoma (B Arrowheads). An IVUS image demonstrated the guidewire passing through the appropriate intraluminal space of the LAD coronary artery. Asterisks indicate the dissected lumen. Arrows indicate the guidewire passing from the LMCA to LCX coronary artery (lower row).

We performed cardiac catheterization with a left transradial approach, and no significant organic stenosis was observed in the coronary arteries (Fig. 1A and B). Next, we performed a coronary spasm provocation test with acetylcholine. During the procedure, a 5Fr JL4 catheter was deeply inserted and the tip of the catheter unexpectedly struck the upper wall of the distal LMCA. The injection of contrast media suddenly caused spiral dissection, which rapidly spread out in the three directions of the proximal LMCA, left anterior descending (LAD) coronary artery, and left circumflex (LCX) coronary artery (Fig. 2A and B Arrows). Coronary flow in both LAD and LCX coronary arteries was interrupted by a coronary hematoma induced by enlargement of the dissected lumen (Fig. 2B Arrowheads). She complained of severe chest pain, and her hemodynamic condition rapidly deteriorated.

**Figure 3:**
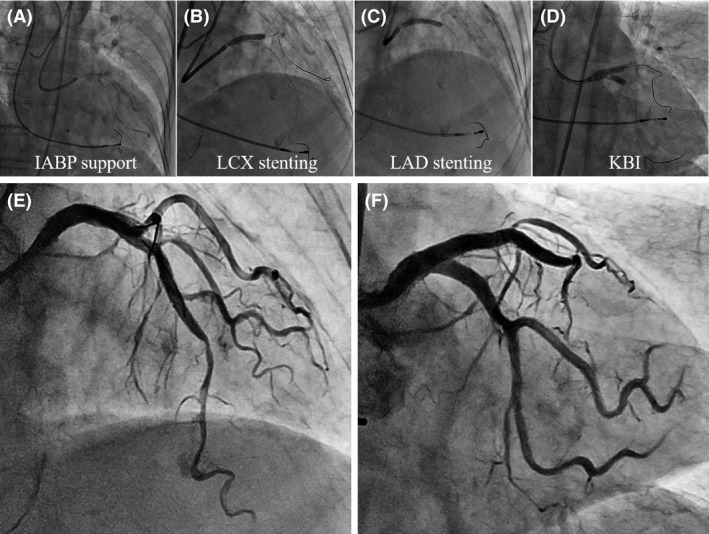
Bailout PCI procedure for the lethal coronary dissection involving LM bifurcation. (A) Guidewires passed in both arteries under IABP support. (B) A DES was deployed from LMCA to the LCX coronary artery. (C) After crushing the proximal segment of the initially deployed DES protruding in LMCA, another DES was deployed from LMCA to the LAD coronary artery. (D) After recrossing a guidewire to the LCX coronary artery, KBI was performed. Final angiography demonstrated sufficient blood flow without delay. (E) Antero‐posterior and cranial view. (F) Antero‐posterior and caudal view.

We immediately switched to bailout PCI. Blood access sites of the bilateral femoral arteries were established. We inserted a 7Fr JL4 guiding catheter into the left coronary artery after setting an intra‐aortic balloon pump (IABP). We successfully passed a Sion guidewire (Asahi Intecc Co., Ltd., Aichi, Japan) into the LCX coronary artery. After checking with intravenous ultrasound (IVUS) that the Sion guidewire was appropriately located inside the vascular lumen, we crossed a Sion blue guidewire (Asahi Intecc Co., Ltd., Aichi, Japan) toward the LAD coronary artery using the Crusade dual lumen microcatheter (KANEKA Corp., Osaka, Japan) (Fig. 3A). The IVUS image demonstrated that the Sion blue guidewire had also passed through the appropriate intraluminal space of the LAD coronary artery (Fig. 2 lower row).

Then, we first deployed a 3.5/18‐mm Resolute Integrity (Medtronic Inc., Minneapolis, MN) drug‐eluting stent (DES) from LMCA to the LCX coronary artery (Fig. 3B), and crushed the proximal segment of the stent protruding in LMCA by inflating a 4.0/15‐mm balloon catheter from LMCA to the LAD coronary artery. Next, we deployed a 3.5/22‐mm Resolute Integrity DES from LMCA to the LAD coronary artery (Fig. 3C). A 3.0/26‐mm Resolute Integrity DES was added in the LAD coronary artery to seal the hematoma, and favorable coronary blood flow was recovered in the LAD coronary artery. We successfully recrossed a Sion black guidewire (Asahi Intecc Co., Ltd.) to the LCX coronary artery and performed kissing balloon inflation (KBI) (Fig. 3D). An IVUS image showed favorable expansion of the stents in both arteries, and final angiography demonstrated sufficient blood flow without delay (Fig. 3E and F). After the procedure, no myocardial damage was observed (Max creatine phosphokinase: 132 U/L), and myocardial contractility was well preserved (Left ventricular ejection fraction: 62%). She could be safely discharged from our hospital after cardiac rehabilitation.

## Discussion

We encountered a patient who suffered from catheter induced sudden lethal coronary dissection involving the left main (LM) bifurcation during CAG with a provocation study to detect coronary vasospasm. This kind of iatrogenic coronary dissection is a rare complication but can become lethal unless it is treated appropriately 3, 4.

The first important step to treat it is to cross a guidewire accurately inside the vascular lumen. Collapse of the vascular lumen, which is induced by enlargement of the dissected lumen, is a critical obstacle to guidewire passage. Expansion of the dissected lumen is usually caused by the injection of contrast media. In our case, although the mechanical injection of contrast media caused the marked enlargement of the dissected space, it was very fortunate that we could successfully introduce a guidewire through the intraluminal LCX coronary artery. After that, the dual‐lumen microcatheter was very helpful to select the optimal intraluminal route of the LAD coronary artery. Thereafter, we confirmed the guidewire passing through the optimal route in both the dissected LAD and LCX coronary arteries.

Next, we had to perform bailout stenting for the dissected coronary arteries involving the LM bifurcation as quickly as possible. Although there have been discussions for a long time about the optimal stenting strategy for bifurcated lesions since the dawning of the coronary intervention era 5, 6, prompt decision‐making was needed at that time. Coronary dissection in this case originated from the upper wall of the distal LMCA and rapidly spread out in three directions of the proximal LMCA, LAD, and LCX coronary arteries. According to the principle of treating coronary dissection, sealing the entry point of the dissection is essential and sufficient. Simply deploying a single stent from LMCA to the LAD coronary artery can be of merit. However, in this case, concern remained that the coronary hematoma in the proximal LCX artery may cause flow limitations. Therefore, we had to consider the validity of a two‐stent strategy.

In the provisional T‐stenting strategy after initial stent deployment in the LAD coronary artery, guidewire recrossing to the LCX coronary artery is required. However, this guidewire maneuver is time‐consuming and the operator may fail to recross the guidewire due to the hematoma in the proximal LCX coronary artery. A V‐stenting strategy is the quickest and easiest procedure in this case. However, there are no data on the safety and efficacy of this strategy in the chronic phase. In the situation of elective PCI, we often adopt a culotte stenting strategy in true bifurcated lesions involving LM bifurcation. However, this procedure is very complex and is considered to be unsuitable in this kind of emergent situation.

We immediately concluded that a crush stenting strategy was the most suitable for the situation. This was because the strategy is easy to perform and we can securely treat the dissection and hematoma in both the LAD and LCX coronary arteries 7. On the other hand, it has been well established that final kissing balloon inflation (KBI) is important to avoid stent distortion and improve short‐ and long‐term angiographic, procedural, and clinical event‐free outcomes in the two‐stent strategy for true bifurcated lesions 8. Also, it has been reported that the absence of final KBI is correlated with a significantly higher adverse event rate compared with the practice of final KBI 9, 10. In this case, we managed to pass a guidewire to the incarcerated LCX coronary artery through double‐folded stent struts and performed final KBI. It took several attempts to achieve this step, but we could perform it without haste, because two‐stent deployment with the method of crush stenting had already recovered favorable coronary blood flow and a stable hemodynamic condition.

## Conclusion

Here, we demonstrated a successful bailout stenting procedure for catheter‐induced lethal coronary dissection involving LM bifurcation. The crush stenting strategy led to a quicker, safer, and more satisfactory outcome. This type of dissection is a rare complication related to coronary interventions, but interventional cardiologists have to pay attention to the risk of complications and prepare for determining the best treatment strategy to overcome the situation. Above all, we must do our best to avoid causing such complications.

## Authorship

HK: involved in study conception, design, drafting manuscript, and the chief doctor for the patient; TN: involved in drafting manuscript and the corresponding author. YH: performed data acquisition. KY: performed data acquisition. DM: performed analysis and interpretation of data. RU: is one of the main doctors. TS: is one of the main doctors. MK: involved in study conception and design, and made critical revision for this report. NK: is a supervisor and one of the main doctors. TT: made critical revision for this report.

## Conflicts of Interest

All authors do not have any conflict of interest.
